# Verapamil inhibits Kir2.3 channels by binding to the pore and interfering with PIP_2_ binding

**DOI:** 10.1007/s00210-022-02342-z

**Published:** 2022-11-29

**Authors:** Panagiotis Xynogalos, Ann-Kathrin Rahm, Sebastian Fried, Safak Chasan, Daniel Scherer, Claudia Seyler, Hugo A. Katus, Norbert Frey, Edgar Zitron

**Affiliations:** 1 Department of Cardiology, Medical University Hospital Heidelberg, Im Neuenheimer Feld 410, 69120 Heidelberg, Germany; 2 DZHK (German Centre for Cardiovascular Research), Partner Site Heidelberg/Mannheim, Heidelberg, Germany; 3 Department of Anesthesiology, Medical University Hospital Heidelberg, Heidelberg, Germany

**Keywords:** Verapamil, Kir channels, IK1 current, Potassium channels

## Abstract

The inwardly rectifying potassium current of the cardiomyocyte (I_K1_) is the main determinant of the resting potential. Ion channels Kir2.1, Kir2.2, and Kir2.3 form tetramers and are the molecular correlate of macroscopic I_K1_ current. Verapamil is an antiarrhythmic drug used to suppress atrial and ventricular arrhythmias. Its primary mechanism of action is via blocking calcium channels. In addition, it has been demonstrated to block I_K1_ current and the Kir2.1 subunit. Its effect on other subunits that contribute to I_K1_ current has not been studied to date. We therefore analyzed the effect of verapamil on the Kir channels 2.1, 2.2, and 2.3 in the Xenopus oocyte expression system. Kir2.1, Kir2.2, and Kir2.3 channels were heterologously expressed in Xenopus oocytes. Respective currents were measured with the voltage clamp technique and the effect of verapamil on the current was measured. At a concentration of 300 µM, verapamil inhibited Kir2.1 channels by 41.36% ± 2.7 of the initial current, Kir2.2 channels by 16.51 ± 3.6%, and Kir2.3 by 69.98 ± 4.2%. As a verapamil effect on kir2.3 was a previously unknown finding, we analyzed this effect further. At wash in with 300 µM verapamil, the maximal effect was seen within 20 min of the infusion. After washing out with control solution, there was only a partial current recovery. The current reduction from verapamil was the same at − 120 mV (73.2 ± 3.7%), − 40 mV (85.5 ± 6.5%), and 0 mV (61.5 ± 10.6%) implying no voltage dependency of the block. Using site directed mutations in putative binding sites, we demonstrated a decrease of effect with pore mutant E291A and absence of verapamil effect for D251A. With mutant I214L, which shows a stronger affinity for PIP_2_ binding, we observed a normalized current reduction to 61.9 ± 0.06% of the control current, which was significantly less pronounced compared to wild type channels. Verapamil blocks Kir2.1, Kir2.2, and Kir2.3 subunits. In Kir2.3, blockade is dependent on sites E291 and D251 and interferes with activation of the channel via PIP_2_. Interference with these sites and with PIP_2_ binding has also been described for other Kir channels blocking drugs. As Kir2.3 is preferentially expressed in atrium, a selective Kir2.3 blocking agent would constitute an interesting antiarrhythmic concept.

**Supplementary Information:**

The online version contains supplementary material available at 10.1007/s00210-022-02342-z.

## Introduction

Among the potassium currents of the heart, the inwardly rectifying current I_K1_ is the main determinant of the resting potential. It is active during the late phase 3 and during the phase 4 of the action potential (Hibino et al. 2010).

Kir2.1 channel subunit together with Kir2.2 and Kir2.3 are the molecular correlates of cardiac I_k1_ current (Nerbonne and Kass 2005). Kir2.1, 2.2, and 2.3 form heterotetramers. Four Kir subunits are necessary for the assembly of one channel pore. There is evidence of regional dispersion of expression of these subunits in different parts of the heart. Kir2.1 has been shown to be expressed in the whole myocardial tissue, whereas Kir2.3 is predominantly expressed in the atria (Gaborit et al. 2007).

I_k1_ current is of particular interest as it has been involved in the pathogenesis of arrhythmias (Nattel et al. 2007). In both simulation and experimental models, an increase of I_k1_ currents has been demonstrated to attenuate the occurrence of focal arrhythmia and provoke reentry based arrhythmias (Noujaim et al. 2007). An overexpression of Kir2.1 has been demonstrated to be a part of the electrical remodeling that occurs in the setting of atrial fibrillation (Luo et al. 2013, Girmatsion et al. 2009). A gain of function mutation in human Kir2.1 channel has been identified in a genetic syndrome of familial atrial fibrillation (Xia et al. 2005).

On the other hand, a reduction of I_k1_ current has also been shown to contribute to arrhythmias. A reduction of I_k1_ current has been shown to destabilize the resting potential and induce delayed afterdepolarizations (Nattel et al. 2007; Myles et al. 2015). In the setting of heart failure, a reduction of I_k1_ current in ventricular cells has been demonstrated (Myles et al. 2015). Genetic syndromes Andersen-Tawil syndrome and catecholaminergic polymorphic ventricular tachycardia, both correlated to ventricular arrhythmias, have been linked to mutations of Kir2.1 channels and subsequent dysfunction of I_k1_ current (Tristani-Firouzi and Etheridge 2010, Anumonwo and Lopatin 2010).

Verapamil is a common drug used to treat arrhythmias (Kato et al. 2004; Reynolds et al. 2005). It has a significant clinical role in frequency control of tachycardic atrial fibrillation (Kirchhof et al. 2016) and for some forms of ventricular tachycardia (Nogami 2011).

Verapamil primarily acts via blocking calcium channels. It has been shown to block both l- and t-type calcium channels (Bergson et al. 2011, Freeze et al. 2006). The main antiarrhythmic action of the drug depends primarily on blocking the calcium current of the atrioventricular (AV) node. This block causes reduced conduction velocity of the AV node, therefore slowing down the conduction of atrial fibrillation on the ventricles (Singh et al. 1982). In an experimental study where atrial fibrillation was induced by rapid pacing in patients, pretreatment with verapamil was shown to attenuate the remodeling effects of atrial fibrillation, an effect correlated to interference with calcium loading of atrial cells (Daoud et al. 1997).

In addition to the calcium current blocking properties, verapamil blocks other potassium channels: it has been demonstrated to reduce I_kur_ (but not I_to_) in human atrial cells (Gao et al. 2004). Jones et al. (2000) demonstrated a reduction of I_K1_ current in ex vivo guinea pig ventricular myocardial cells after incubation with verapamil.

Verapamil has been demonstrated to block cardiac potassium channels in experimental settings: it has been shown to block HERG channels expressed in HEK-293 cells (Zhang et al. 1999; Duan et al. 2007) and KATP channels (Ninomiya et al. 2003) expressed in COS7 cells. In Xenopus oocytes expressing most potassium channels of the heart simultaneously, verapamil was shown to block Kir2.1 channel current (current reduction at 63.9 ± 2.4% of the initial current) as well as HERG and KvLQT1/IsK channels (Waldegger et al. 1999).

As verapamil has been demonstrated to reduce I_K1_ current and block Kir2.1 channels, we examined the effect of verapamil on all Kir subunits (2.1, 2.2, and 2.3) constituting the cardiac I_K1_ current comparatively in a heterologous expression system. Furthermore, we provide experimental data to delineate the molecular basis of the effect of verapamil on Kir2.3. We demonstrate that verapamil also blocks Kir2.2 and Kir2.3 channels in a heterologous expression system. We further elucidate the properties of block on Kir2.3 channels (voltage independence). Moreover, by generating channel mutants in putative binding sites already described, we demonstrate dependence of block on sites E291 and D251 as well as dependence on PIP_2_ binding.

## Materials and methods

### Preparation of DNA and expression

Kir2.1, 2.2, and 2.3 DNA is a friendly contribution of Dr. Barbara Wible (Cleveland, OH, USA) and Dr. Carol Vandenberg (Santa Barbara, CA, USA). Complementary RNA was synthesized from these DNA by using mMESSAGE mMASHINE. For the Kir2.1 and Kir2.2 constructs, T7 polymerase was used, whereas for Kir2.3 T3 polymerase was used. The Kir2.3 mutants that were used for the experiments were constructed by introducing single point mutations by using the QuikChangeTM site-directed mutagenesis kit (Stratagene, La Jolla, USA) as previously described (Karle et al. 2002). The confirmation of the correct mutations was performed with direct DNA sequencing (SeqLab, Göttingen, Germany).

Expression of the constructed cRNAs was performed in *Xenopus laevis* oocytes. Stage V and VI defolliculated oocytes were injected with cRNA of concentration 50–5000 ng/µl (50 nl per oocyte). One to 3 days after injection, experiments were performed.

All performed experiments conform to the Guide for the Care and Use of Laboratory Animals 8th edition, revised 2011.

### Electrophysiological recording

The technique used for current measurement is the two microelectrode voltage-clamp technique in *Xenopus laevis* oocytes. The data were filtered at 1 to 2 kHz (− 3 dB four-pole Bessel filter) prior to digitalization. No leak subtraction was performed during the experiments. Recordings were performed using a commercially available amplifier (Warner OC-725A, Warner Instruments, Hamden, USA) and pCLAMP software (Axon Instruments, Foster City, USA).

### Data analysis

Statistical data are presented as mean ± standard error of the mean. *N* represents the number of experiments performed. Statistical significance tests used were Student’s *t*-test and one-way ANOVA test, where applicable. Significance level was determined as a *p* value less than 0.05. For the statistical analysis, the software program Origin Pro 8.1 (Origin Lab Corp., Northampton, MA) was used. For the dose response curve, the results at different concentrations were fitted to the Hill equation: *I*/*I*_0_ = *I*_0_/(1 + *X*/*IC*_50_)^*n*^, with *I*/*I*_0_ being the relative current, *I*_0_ the unblocked current amplitude, *X* the drug concentration, *IC*_50_ the concentration for half maximal block, and *n* the Hill coefficient.

### Solutions and drugs

The control solution as well as the solution used for the voltage clamp measurements contained in mM: 5 KCl, 100 NaCl, 1.5 CaCl2, 2 MgCl2, and 10 HEPES. The pH of this solution was corrected to 7.4 with 1 M NaOH. Electrodes were filled with 3 M KCl solution. All experiments were performed in regulated room temperature 20–21 °C.

Verapamil powder was acquired commercially (Sigma Aldrich, Steinheim, Germany). This was initially diluted with dimethyl-sulfoxide (DMSO) to stock solution of 100 mM. This solution was stored at − 20 °C. At the day of the experiments, this solution was further diluted to the desired concentration. The diluter used was the control solution.

For the screening experiments, a concentration of 300 µM was chosen. As the oocyte membrane poses a barrier in the diffusion of substances on the expressed channels in oocyte expression system, higher doses are generally needed. Therefore, as to achieve a maximum effect we chose a high concentration of verapamil. We also oriented the choice to the already measured concentrations for verapamil. For example, Waldegger et al. (1999) measured the effect on Kir2.1 channels with a concentration of 1 mM. Jones et al. (2000) measured an *IC*_50_ of 220 µM of verapamil on Kir2.1 channels.

Xenopus oocytes were incubated in the control solution or in the verapamil solution for 30 min at room temperature. Measurements were performed before and after the incubation.

Further for the wash-in experiments, a continuous infusion of the verapamil solution was introduced in the voltage clamp bench. For the wash-out experiments, the infusion was changed to control solution.

## Results

### Verapamil at a concentration of 300 µM blocks all cardiac Kir channels and the effect is most pronounced on Kir2.3 channels

After heterologous expression of Kir2.1, 2.2, and 2.3 respectively, a voltage command protocol was applied. From a holding potential of – 80 mV, a voltage command was applied for 400 ms starting from the hyperpolarized potential of − 120 mV and incrementally increased at steps of 10 to + 40 mV. The measurements were performed once before and after incubation with control solution for 30 min or verapamil 300 µM for 30 min. Inward current amplitudes at − 120 mV were compared before and after incubation for each oocyte, and the change to the initial current was calculated.

With the oocytes expressing Kir2.1 channels, when incubated with control solution we measured a discrete current increase of 12.3 ± 2.7% (*N* = 7) compared to the initial current. After incubation with 300 µM verapamil, we measured a significant reduction by 41.36% ± 4.2 of the respective initial current (*N* = 5) (*p* = 5.01 × 10^−7^).

The oocytes expressing Kir2.2 channels displayed an increase by 22.8 ± 4.9% of the initial current (*N* = 5) after incubation with the control solution. The verapamil effect was apparent, but reduced when compared with the effect on Kir2.1 channels. We measured a reduction by 16.51 ± 3.6% (*N* = 9) of the initial current after incubation with the verapamil solution (*p* = 3.57 × 10^−5^).

**Fig. 1:**
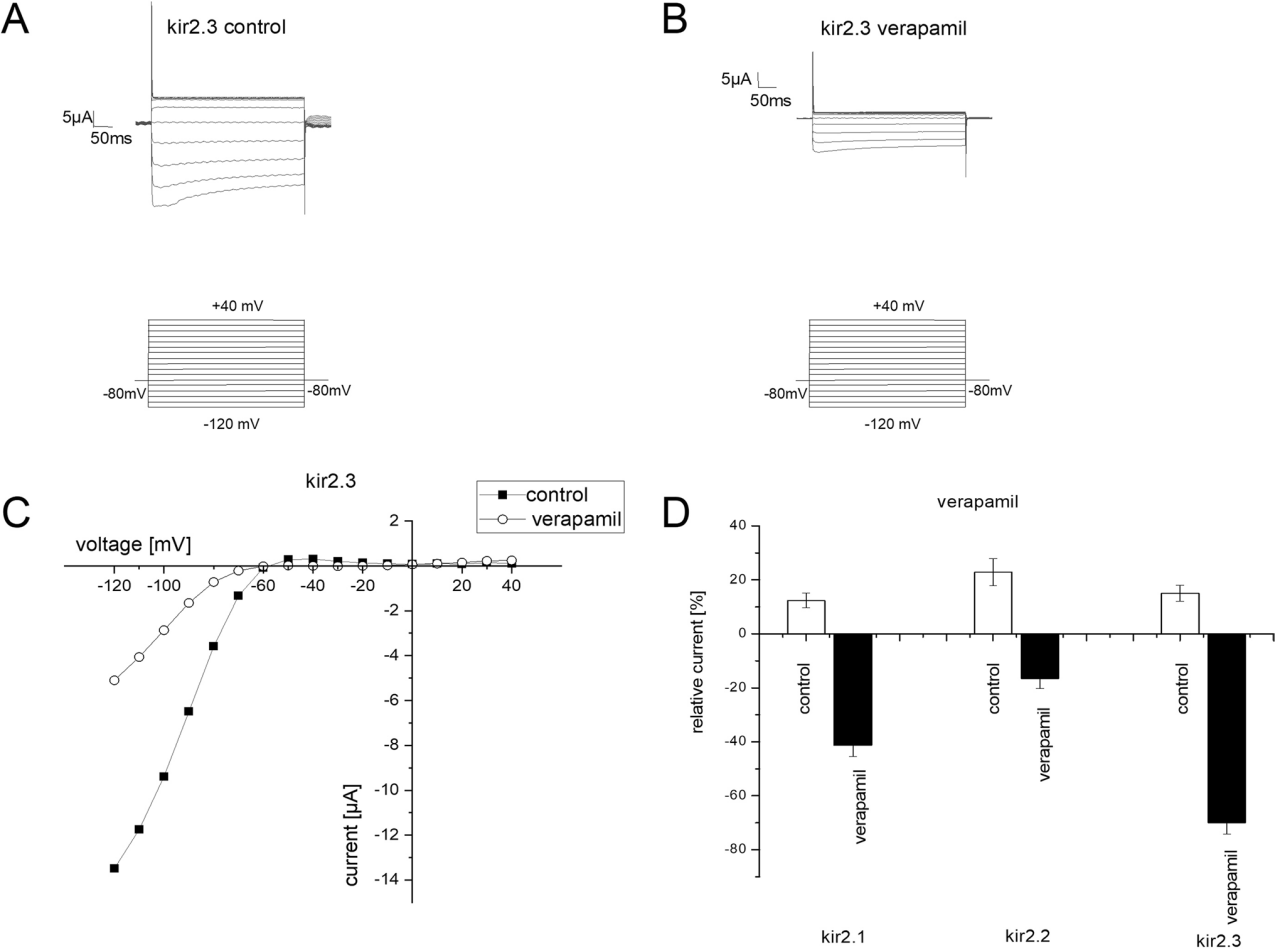
**A** and **B** Representative current traces of current produced along with the voltage clamping protocol before (**A**) and after (**B**) incubation in verapamil. **C** The current–voltage relationships before and after verapamil application. **D** Overview of the current change from baseline for Kir2.1, 2.2, and 2.3 at − 120 mV in control solution and verapamil solution, respectively. For Kir2.1 expressing oocytes, verapamil causes a current reduction by 41.36% ± 4.2% (*N* = 7) of the initial current. With Kir2.2 expressing oocytes, the current reduction is 16.51 ± 3.6% (*N* = 9) of the initial current. In Kir2.3 expressing oocytes, the current reduction reached 69.98 ± 4.2% of the initial current (*N* = 7). In control solution, the current showed a small increase compared to the initial current of 12.3 ± 2.7% (*N* = 7) for Kir2.1, 22.8 ± 4.9% for Kir2.2, and 15 ± 2.9% for Kir2.3

With Kir2.3 channels, the effect was more pronounced. Under control conditions, Kir2.3 channels showed an increase of 15 ± 2.9% of the initial current (*N* = 10), whereas after verapamil incubation we observed a marked reduction by 69.98 ± 4.2% of the initial current (*N* = 7) (*p* = 3.45 × 10^−11^). In Fig. 1, we summarize these findings (supplementary Fig. 1 and 2, supplement1.xls).

### Concentration-dependent inhibition of Kir2.3 currents

**Fig. 2:**
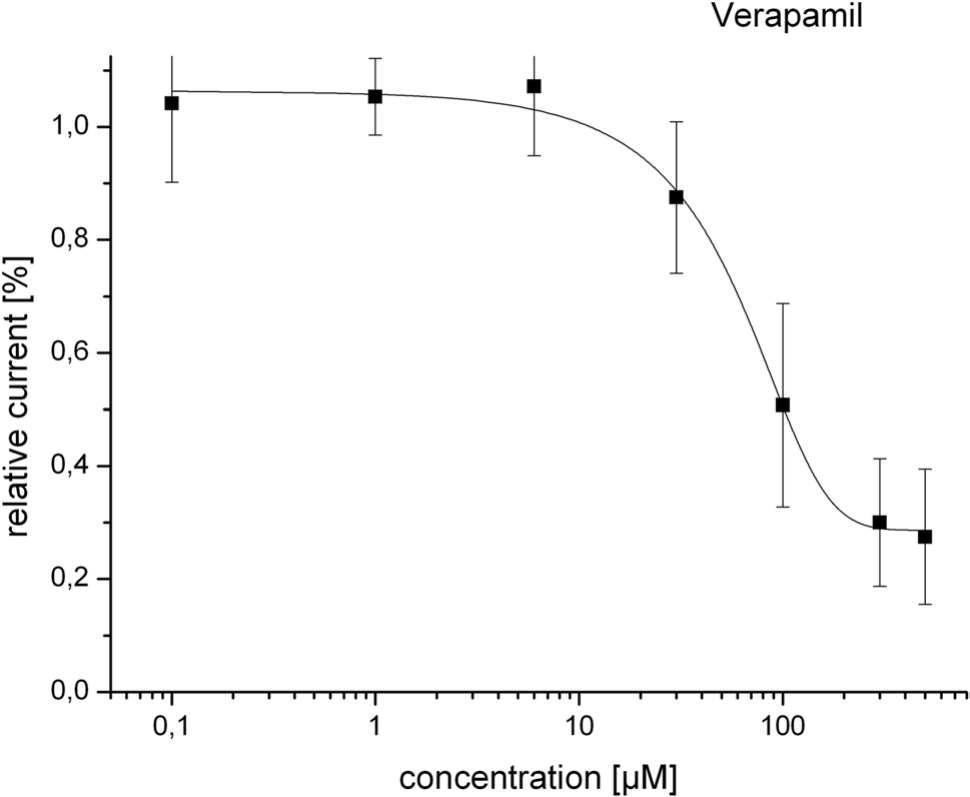
Dose response curve of Kir2.3 channels at different verapamil concentrations. After fitting the results to the Hill equation, we obtained an *IC*_50_ of 58.10 µM which represents a low affinity of the drug in the oocyte expression system

As the effect was most prominent on Kir2.3 channels, we focused the further analysis on these channels. We therefore measured the effect of verapamil at different concentrations. Kir2.3 expressing oocytes were incubated for 30 min either in verapamil at the respective concentration or in control solution for 30 min. The currents were measured as a % change normalized to the current after incubation for the same time in control conditions. At a concentration of 0.1 µM verapamil, we measured no effect with the normalized current unchanged at 104.2 ± 4.9% (*N* = 6). At concentration of 1 µM, no effect was observed either with a normalized current at 105.3 ± 3.02% (*N* = 6). At a substance concentration of 6 µM, no effect was observed with a respective normalized current of 107 ± 4.6% (*N* = 6). At a concentration of 50 µM, there was a reduction to 87.5 ± 5% (*N* = 6) of the control current, whereas at a concentration of 100 µM there was a reduction to 50.8 ± 5.4% (*N* = 5). At a concentration of 150 µM, the current was reduced to 30 ± 4.2% (*N* = 6), and at 300 µM the current was reduced to 27.5 ± 4.8% (*N* = 6). These results were then fitted to the Hill equation to obtain the concentration/effect relation. This result is shown as plot on Fig. 2. The results yielded an *IC*_50_ of 58.10 µM, which represents a low affinity of the drug on the channel in this expression system (supplement2.xls).

### The verapamil effect initiates rapidly and is only partially reversible upon wash-out

**Fig. 3:**
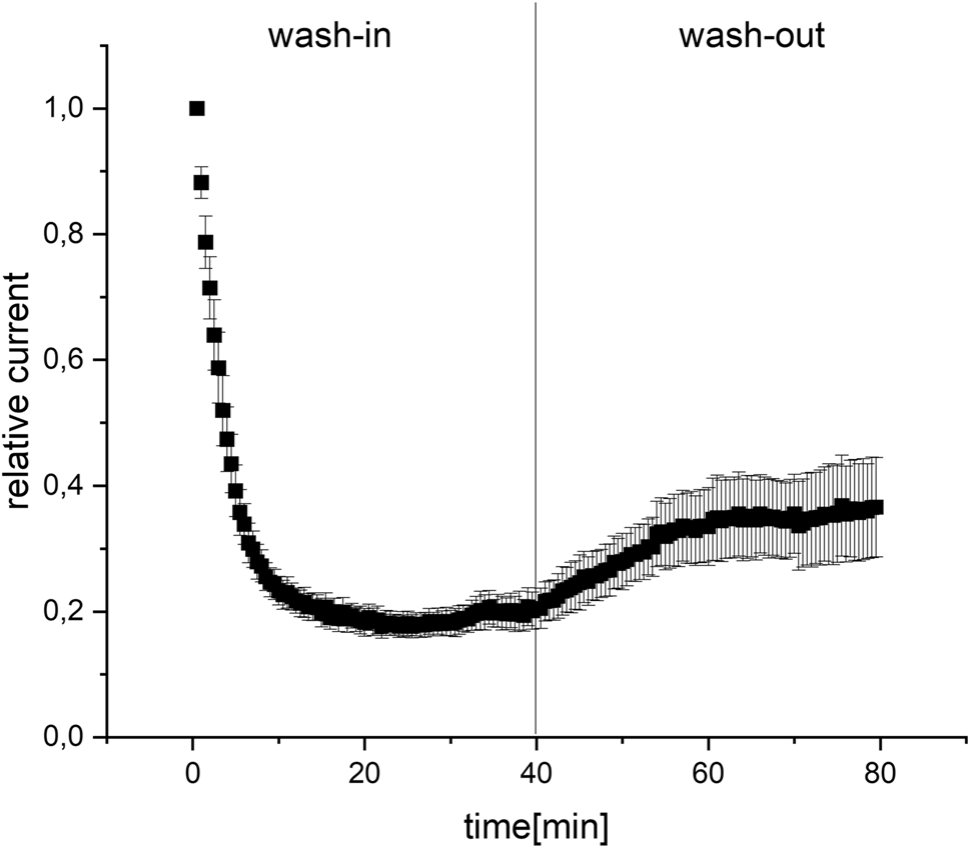
Wash-in: relative current change over time after verapamil infusion in the bath solution. At intervals of 5 s, a voltage command to − 120 mV was given and the inward currents were measured. A steady state inhibition is seen after approximately 20 min (relative current of 25.8 ± 3% of the initial current). Wash-out: relative current change over time after change of the infusion of the bath solution with control solution. We observe a partial reversal of verapamil effect to a level of 46.09 ± 7% relative to the initial current

To further elucidate the blocking properties, we applied a wash-in and wash-out protocol. Oocytes expressing Kir2.3 channels were clamped at a holding potential of − 80 mV in control solution. After that an infusion of 300 µM verapamil was initiated (wash-in). At intervals of 5 s, a voltage command to − 120 mV was given and the inward currents were measured. After the maximum current reduction was observed in form of a steady state of current, the infusion was changed again to control solution (wash-out) and the same voltage command protocol was applied. These data are shown in Fig. 3. After 20 min, we observed the maximal effect of the verapamil infusion with a relative current of 25.8 ± 3% of the initial current, so we conclude that the effect initiates relatively quickly. At the wash-out phase, we observed only a slight recovery of the current to a level of 46.09% ± 7 relative to the initial current (*p* = 0.03). We therefore concluded only a partially reversible verapamil effect (*N* = 6, Fig. 3, supplement3.xls).

### The block is not voltage dependent

**Fig. 4:**
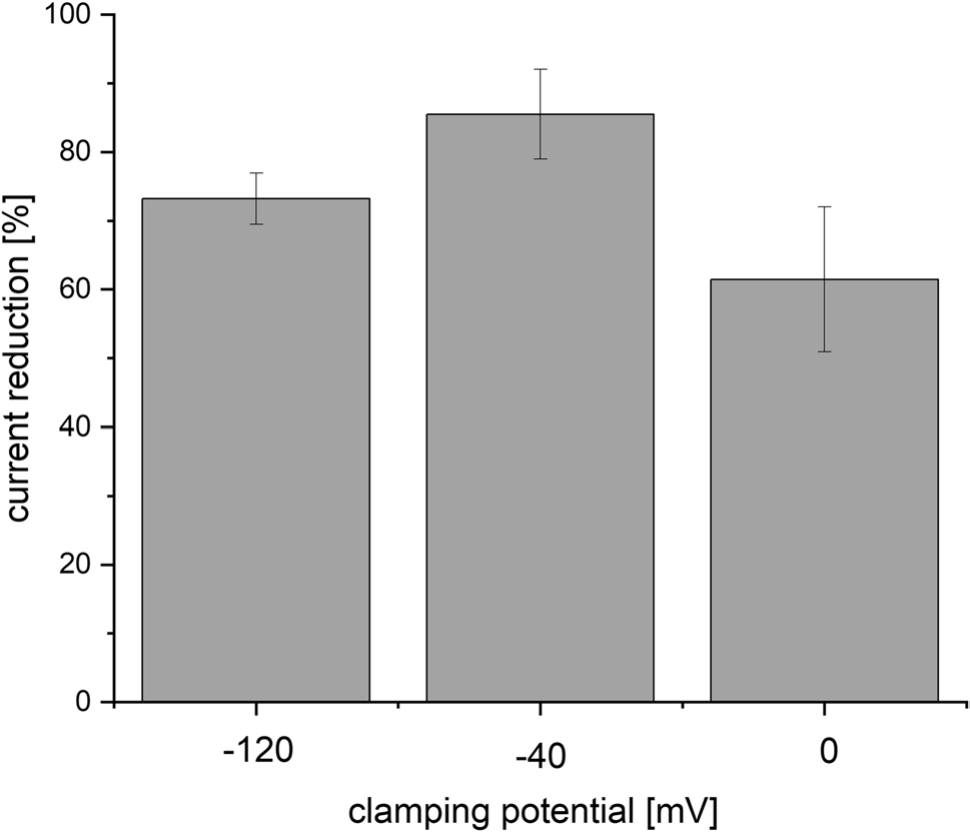
Amount of block of verapamil on Kir2.3 channels at different clamping voltages. There was no significant difference at the amount of block at clamping potentials − 120 mV, − 40 mV, and 0 mV with current reduction (relative to the initial current) of 73.2 ± 3.7%, 85.5 ± 6.5%, and 61.5 ± 10.6%, respectively. ANOVA *F* value 2.58, *p* = 0.11

Further we measured the effects of verapamil at different clamping potentials at − 120, − 40, and + 0 mV. We observed a reduction of the current of 73.2% ± 3.7 (*N* = 6) at − 120 mV, 85.5 ± 6.5% at − 40 mV, and 61.5 ± 10.6% at 0 mV. The ANOVA test showed no significant difference at these potentials (*p* = ns for the comparison). Hence, the block is not voltage dependent. The results are summarized in Fig. 4 (supplement4.xls).

### Delineating the blocking mechanism-pore mutants

A well described mechanism of block of Kir2.1 channels is via electrostatic interaction with residues forming the inner lining of the pore. These sites include for Kir2.1 channels, among other residues E224, F254, D259, and E299. These negatively charged amino acids serve as residues for electrostatic interaction with drugs. Drug binding in these regions narrows the channel pore and blocks the flow of potassium ions. This is the case for drugs chloroquine (Rodríguez-Menchaca et al. 2008; Noujaim et al. 2010) and pentamidine (de Boer et al. 2010). Equivalent residues on Kir2.3 channels have been identified via comparative alignment and have been used to screen for putative binding sites of quinidine on Kir2.3 channels (Koepple et al. 2017; de Boer et al. 2010). These residues include E291, D251, E216, and D247. Pore mutants E291A, D251A, E216A, and D247A (Koepple et al. 2017) were used to scan for putative molecular determinants of quinidine block.

Equivalently we generated pore mutants E291A, D251A, E216A, and D247A of Kir2.3 channels and measured the effect of verapamil as compared to the effect on wild type channels. The same voltage protocol was applied as with wild type channels. The currents were normalized to the control current for the comparison.

With pore mutant E291A (*N* = 6 for verapamil and *N* = 8 for controls), we observed a decrease of the verapamil effect compared to the wild type current. Normalized current was reduced to 27.05 ± 0.04% of the control current in wild type channels (*N* = 19 for control experiments and *N* = 6 for verapamil incubation) after verapamil treatment, whereas in mutant E291A it was reduced only to 73.46 ± 0.08% of the control current (*p* = 3.03 × 10^−4^).

With mutant D251A, we observed an abolishment of verapamil effect. Here the normalized current was minimally reduced to 94.9 ± 0.06% (*N* = 7 for verapamil and *N* = 4 for control) (*p* = 3.48 × 10^−7^ in comparison to the wild type channels). So we can conclude that these pore residues (E291 and D251) seem to be essential for the verapamil effect.

**Fig. 5:**
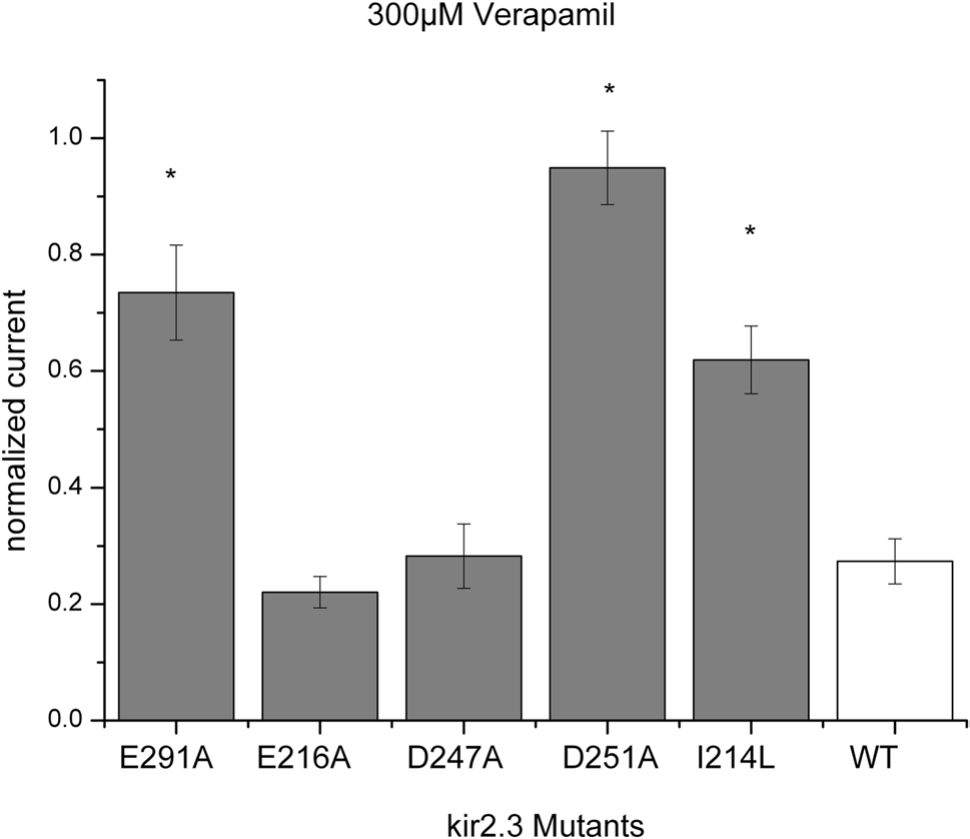
Verapamil effect (displayed as normalized current) of different Kir2.3 mutants compared to wild type channels. The effect on mutants E216A and D247A was similar to wild type (WT) channels. On pore mutants E219A and D251A, there is a reduction of verapamil effect. The effect of verapamil is also reduced on PIP_2_ mutant I214L. * marks statistical significance

On the contrary the effect of verapamil was not different among wild type, D247A (*N* = 7 for verapamil and *N* = 8 for controls), and E216A (*N* = 7 for verapamil and controls respectively) mutant channels. The relative effect was 28.2 ± 0.06% with mutant D247A and 22.05 ± 0.02% with mutant E216A (*p* = ns for each comparison). The results are summarized in Fig. 5 (supplement6.xls).

### PIP_2_interference

Another mechanism of block on Kir2.3 channels is interference with regulating PIP. These phospholipids are essential for channel function probably by stabilizing the open conformation. For example, the drug mefloquine and carvedilol have been shown to interfere with Kir2.3 channel-PIP_2_ interaction, thus reducing the current. Mutant channel I214L was used to delineate this effect (Ferrer et al. 2011). For this mutant, a stronger PIP_2_ affinity has been described. If an involvement of PIP_2_ interference mechanism of verapamil action is present, we would expect a weaker effect of verapamil with this mutant (Du et al. 2004).

We therefore also generated the same mutant and compared the effect of verapamil on oocytes expressing the mutant channel and oocytes expressing WT channels. The oocytes were incubated in a bath solution of 300 µM verapamil or control for 30 min as described above. Measurements were performed before and after incubation. The voltage protocol applied was the same as aforementioned (incremental voltage steps from − 120 to + 40 V, holding potential − 80 mV). The measurements were made on the maximal inward current at − 120 mV. The currents were normalized to the control current for the comparison. In this case, the observed effect was indeed less pronounced. I214L mutant channels displayed a normalized current reduction to 61.9 ± 0.06% (*N* = 7 for verapamil and *N* = 14 for controls) of the control current, whereas wild type channels had a reduction of 27.36 ± 0.04% (*p* = 0.04 compared to the control current). Results are also shown in Fig. 5 (supplement6.xls).

## Discussion

Verapamil is a widely used substance for rate control of atrial fibrillation (Kirchhof et al. 2016). There is evidence of a verapamil effect on I_K1_ current in addition to its effects on Ca current, I_Kr_ and I_Ks_ current. This effect however was until now not evaluated on the specific channel subunits. Jones et al. (2000) reported a reduction of I_K1_ current in guinea pig ventricular cardiomyocytes, whereas Waldegger et al. (1999) reported a significant reduction of current produced by Kir2.1 subunits expressed in *Xenopus laevis* oocytes where other cardiac potassium channels were coexpressed including the potassium channels Kv1.1, Kv1.5, Kir2.1, and HERG, and the IsK subunit of the I_Ks_-channel complex. Contrary to these findings in a rabbit atrium tachycardia pacing model, no effect of verapamil pretreatment on I_K1_ was reported by Laszlo et al. (2007). We therefore oriented our study towards the effects of verapamil on all the Kir channel subunits that constitute the cardiac I_K1_ current (Kir2.1, 2.2, and 2.3). In this study, we observe a strong effect of verapamil on the Kir2.3 subunits whereas a smaller effect on the Kir2.1 subunit and an even smaller effect on the Kir2.2 subunit were present.

There are other drugs showing preferential block of Kir2.3 subunits. For example, carvedilol has been demonstrated to block Kir2.3 channels to a much larger extent than Kir2.1 channels (Ferrer et al. 2011). Quinacrine has been demonstrated to inhibit Kir2.3 more than Kir2.1 channels expressed in HEK 293 cells (López-Izquierdo et al. 2011a, b). Propafenone is also a more potent blocker of Kir2.3 compared to both Kir2.1 and Kir2.2 as demonstrated by Amorós et al. (2013).

The block initiates relatively quickly. We observed a steady state within minutes of infusion with verapamil in our experiments. Equivalently carvedilol has been also demonstrated to block Kir2.3 expressed in HEK cells relatively rapidly within minutes of application (Ferrer et al. 2011). Contrary to this, dronedarone has been shown to block Kir2.1 subunits more slowly (Xynogalos et al. 2014), possibly due to these molecules being less hydrophilic than verapamil and diffusing more slowly intracellularly.

The *IC*_50_ was concordant with a moderate affinity of binding. Along the measured clamping potentials, we saw no voltage dependence of block. Macroscopic current data shown by Jones et al. (2000) however demonstrate a lesser extent of verapamil block of the outward component of the I_k1_ current in guinea pig cardiomyocytes.

### Mechanism of action

To further delineate the mechanism of block, we generated mutants where putative binding amino acid sites of electrostatic interaction are replaced with neutral amino acids. These sites were identified by means of comparative alignment of Kir2.3 channels to Kir2.1 channels, where interaction sites have been identified and described (Ferrer et al. 2011; López-Izquierdo et al. 2011a, b; Ponce-Balbuena et al. 2009). Indeed these sites have been previously used to delineate the binding sites for quinidine on Kir2.3 channels (Koepple et al. 2017).

Interestingly we observed a diminished verapamil effect on mutant E291A and an abolished verapamil effect on mutant D251A. Since these are the equivalent residues of inner lining of the pore on Kir2.1 channels, this would be in line with the hypothesis of a pore blocking mechanism of action by electrostatic interaction in the cytoplasmic side of the pore of Kir2.3 channels. The same mechanism of block has been described for quinidine (Koepple et al. 2017).

Another described mechanism of pharmacologic action is the interference with activating membrane phosphoinositides. These lipids, particularly PIP_2_, cause conformational changes of the channel leading to transference to the open state (Hansen et al. 2013). Some drugs interfere with this binding thereby preventing the channel from switching to the open state. To examine a possible effect, we produced Kir2.3 mutant I214L. This mutant has a higher affinity to PIP_2_ so that a substance that interferes with its binding would cause a less pronounced effect compared to the wild type channels. Interestingly this was the case with verapamil. Based on our results, a PIP_2_ interfering mechanism is also a possible contributor to the blocking properties of verapamil. An equivalent inhibitory effect on Kir2.3 channels has been proposed for mefloquine (López-Izquierdo et al. 2011a, b) and carvedilol (Ferrer et al. 2011).

In conclusion, verapamil apparently blocks Kir2.3 channels both by intracellular pore block as well as interference with PIP_2_ binding. Interestingly quinacrine has also been demonstrated to have a complex blocking mechanism both interacting with PIP_2_ and blocking the pore (López-Izquierdo et al. 2011a).

### Limitations

We measured a comparatively high *IC*_50_ of 58.1 µM for verapamil on Kir2.3 in the Xenopus oocyte expression model. Equivalent *IC*_50_ values were reported in Xenopus oocytes measurements by Waldegger et al. (1999), who measured an *IC*_50_ for verapamil of 14.0 µM for Kv1.1, 5.1 µM for Kv1.5, 161.0 µM for IKs, and 3.8 µM for HERG channels, respectively. For Kir2.1, no *IC*_50_ was reported in this study, but the concentration used was up to 1 mM. In the above study, however, the coexpression of all above mentioned potassium channels was a limitation. Generally higher *IC*_50_ values for drugs are expected in the Xenopus oocyte expression system partially due to the mechanical barrier from yolk sacks and vitelline membranes (Madeja et al. 1997; Rolf et al. 2000). In guinea pig ventricular myocytes studied by Jones et al. (2000), verapamil caused significant inhibition of I_K1_ current at 30 μM and higher concentrations, and the Hill equation describing the verapamil data showed an *IC*_50_ of 220 µM for the outward component of the current. Contrary to the above measured *IC*_50_ values, higher affinities of verapamil on potassium channels have been described in other experimental models. For instance, verapamil showed an *IC*_50_ of 143.0 nM on HERG channels in native cardiomyocytes (Zhang et al. 1999). Orvos et al. (2019) measured an *IC*_50_ of 268 nM on I_kr_ current in rabbit cardiomyocytes. In a clinical context, the therapeutic concentration of verapamil in patients is 125 to 400 ng/ml which represents 0.2–0.8 µM (McTavish and Sorkin 1989), with 90% of the drug bound on proteins. Therefore, a relevant block of verapamil on Kir2.3 channels in a clinical setting is not certain.

Verapamil consists of two enantiomers with described different attributes regarding ion channel action. For instance, R-verapamil has only 5–10% of the calcium blocking efficacy of the S-enantiomer (Waldegger et al. 1999). The possible differential effect of R- and S-enantiomers was not addressed in this study.

Other known mechanisms of interference with I_k1_ current are changes of the ion transfer on the membrane or change of the ion channel expression levels. Chronic verapamil treatment has been shown to remodel calcium channels. It has been shown to increase Ca(V)1.2 mRNA and protein and to significantly increase I(Ca,L) in myocardial cells of mice (Schroder et al. 2007). Whether an equivalent effect is shown on the expression on Kir channels was not addressed in this study.

As shown in this study, we measured a more pronounced effect of verapamil on Kir2.3 channels as on Kir2.1 channels. It is known that the expression of Kir subunits in the heart is not homogenous with Kir2.3 preferentially expressed in the atrial tissue (Ehrlich 2008). Whether the I_k1_ blocking properties of verapamil in the atrial tissue are more pronounced than in ventricular tissue has not to our knowledge been evaluated yet.

As a reduction of I_k1_ current would destabilize rotors and an increase of I_k1_ current in atrial fibrillation is a known phenomenon (although with no certain contribution to the perpetuation of arrhythmias), verapamil could possibly elicit some effects on the atrial fibrillation itself and not only on the AV node conduction. This hypothesis has not been evaluated to date.

Atrial fibrillation is one of the most common arrhythmias and an atrial specifically acting medication would be of enormous interest. In this context, Kir2.3 specific action would be an interesting theoretical concept as there is an atrial predominance of Kir2.3 expression (Ehrlich 2008). In line with this concept, efforts of producing Kir2.3 selective blockers have already been published, as for instance with substance VU573 (Raphemot et al. 2011).

### Conclusion

Verapamil has been described to block macroscopic I_K1_ current. A verapamil effect on human Kir2.1 channels has also been described in previous studies. In this study, we demonstrate verapamil effect on the other subunits constituting I_K1_ current, Kir2.2 and Kir2.3. The extent of block is larger on Kir2.3 subunits. This block appears to be exerted via a complex mechanism of both drug interaction with the pore and drug interference with PIP_2_ binding, as already described for other Kir blockers.

## Supplementary Information

Below is the link to the electronic supplementary material.

Supplementary file1 (JPG 561 kb)

Supplementary file2 (JPG 518 kb)

Supplementary file3 (XLS 27 kb)

Supplementary file4 (XLS 27 kb)

Supplementary file5 (XLS 88 kb)

Supplementary file6 (XLS 30 kb)

Supplementary file7 (XLS 77 kb)

Supplementary file8 (XLS 33 kb)

## Data Availability

Data and materials are available upon reasonable request. The authors declare that all data were generated in-house and that no paper mill was used.
