# Supplementary figures and images for: Verapamil inhibits Kir2.3 channels by binding to the pore and interfering with PIP2 binding
Source: Naunyn Schmiedebergs Arch Pharmacol. 2022 Nov 29;396(4):659–67. doi: 10.1007/s00210-022-02342-z (PMC10042922; doi:10.1007/s00210-022-02342-z)

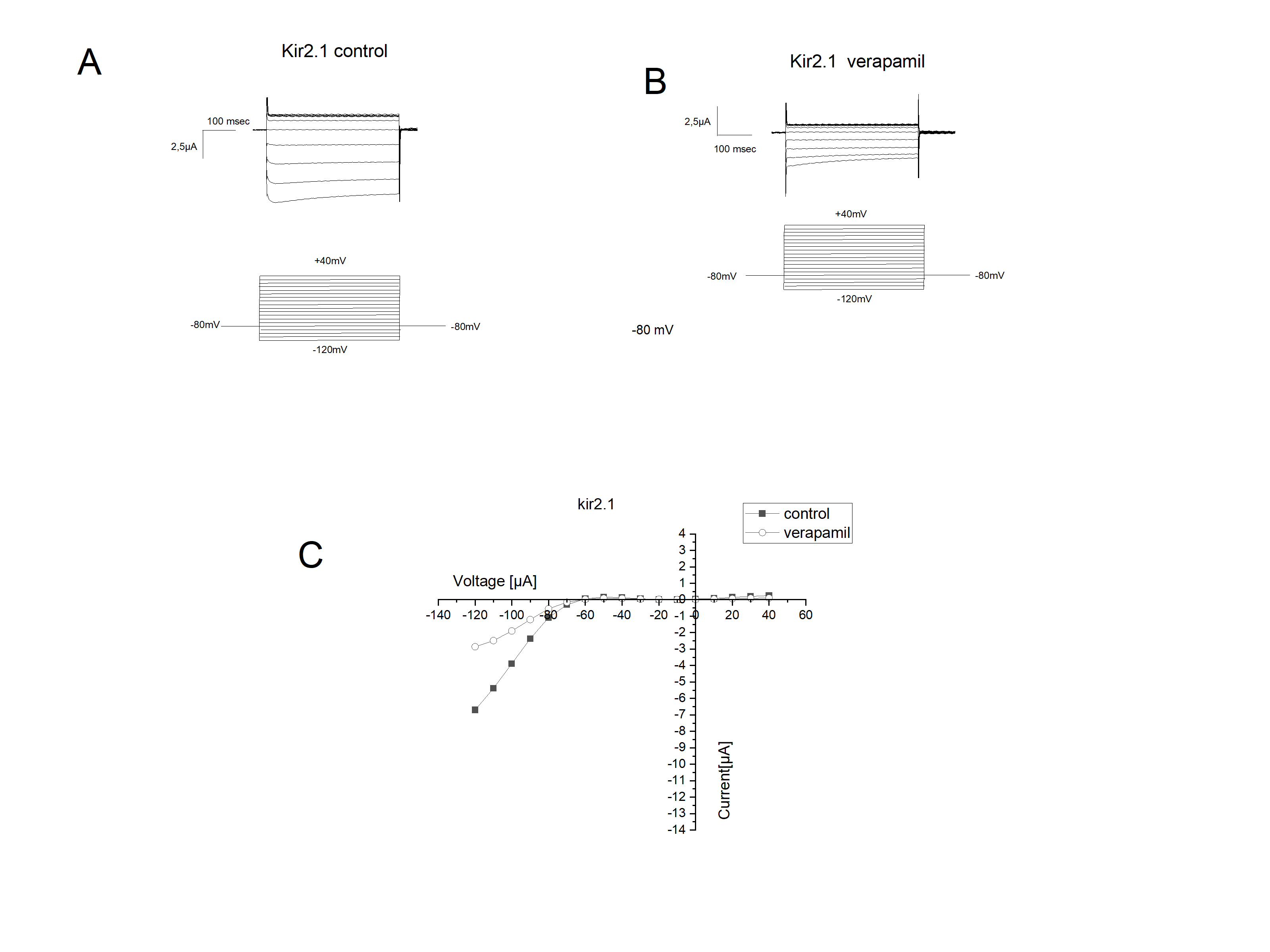

Supplement: Supplementary file 1 — Supplementary file1 (JPG 561 kb) [file 210_2022_2342_MOESM1_ESM.jpg]

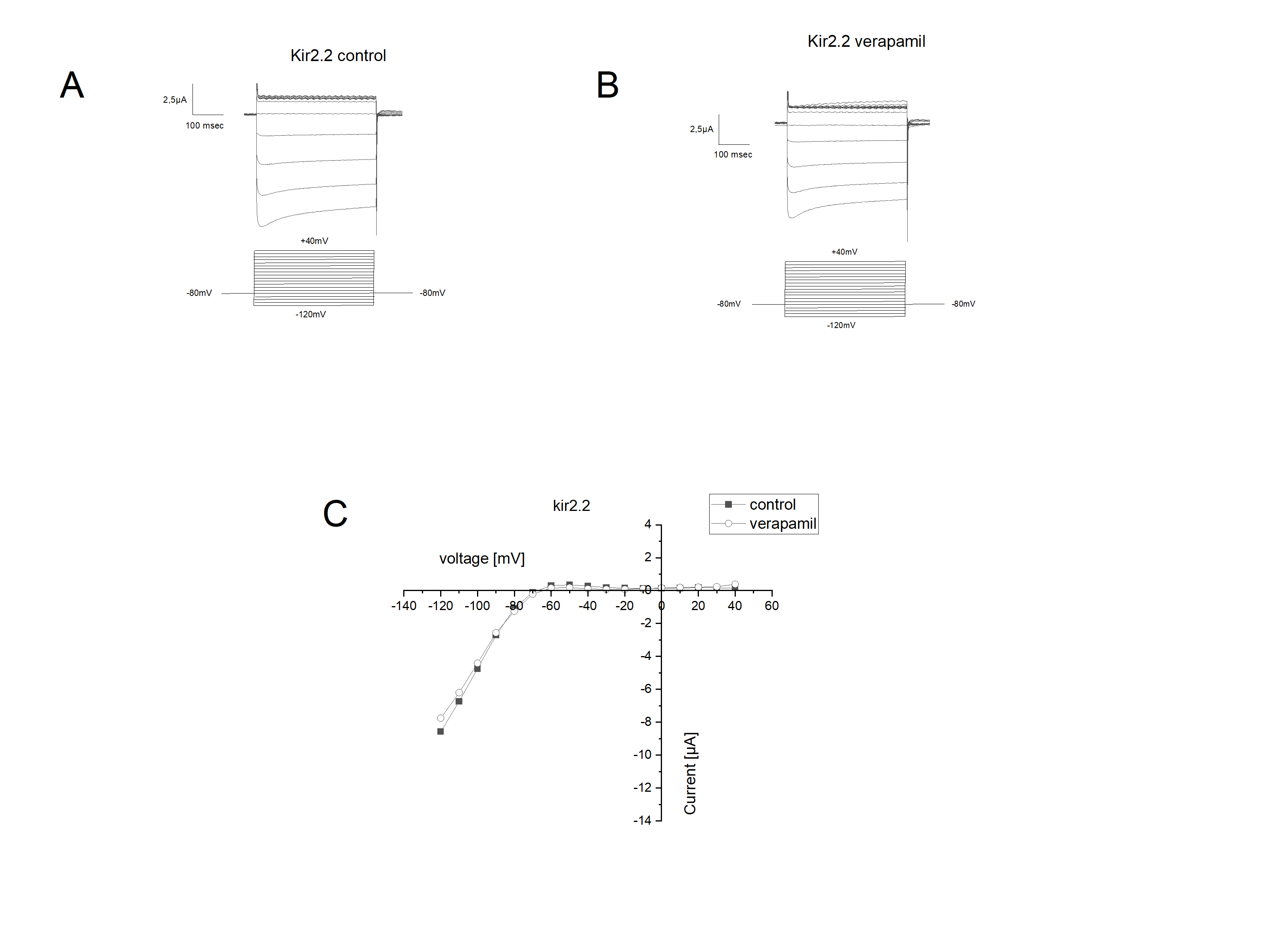

Supplement: Supplementary file 2 — Supplementary file2 (JPG 518 kb) [file 210_2022_2342_MOESM2_ESM.jpg]
